# Association of Personal Care and Consumer Product Chemicals with Long-Term Amenorrhea: Insights into Serum Globulin and STAT3

**DOI:** 10.3390/toxics13030187

**Published:** 2025-03-05

**Authors:** Ziyi Li, Xue Song, Daniel Abdul Karim Turay, Yanling Chen, Guohong Zhao, Yingtong Jiang, Kun Zhou, Xiaoming Ji, Xiaoling Zhang, Minjian Chen

**Affiliations:** 1 State Key Laboratory of Reproductive Medicine, Center for Global Health, School of Public Health, Nanjing Medical University, Nanjing 211166, China; 18851020597@163.com (Z.L.); songxue9922@163.com (X.S.); danielabdulkarimturay91@gmail.com (D.A.K.T.); cyanling9211@163.com (Y.C.); zgh18156184959@163.com (G.Z.); ytjiang61@163.com (Y.J.); zk@njmu.edu.cn (K.Z.); 2 Key Laboratory of Modern Toxicology of Ministry of Education, School of Public Health, Nanjing Medical University, Nanjing 211166, China; 3 Department of Epidemiology, Center for Global Health, School of Public Health, Nanjing Medical University, Nanjing 211166, China; 4 Department of Occupational Medicine and Environmental Health, School of Public Health, Key Laboratory of Public Health Safety and Emergency Prevention and Control Technology of Higher Education Institutions in Jiangsu Province, Nanjing Medical University, Nanjing 211166, China; jxmnjmu@163.com; 5 Department of Hygienic Analysis and Detection, Nanjing Medical University, Nanjing 211166, China; zhangxl3@njmu.edu.cn

**Keywords:** personal care products, long-term amenorrhea, polyfluorinated alkyl substances, mixed exposure, mediation analysis, molecular docking

## Abstract

Chemicals in personal care and consumer products are suspected to disrupt endocrine function and affect reproductive health. However, the link between mixed exposure and long-term amenorrhea is not well understood. This study analyzed data from 684 women (2013–2018 National Health and Nutrition Examination Survey) to assess exposure to eight polyfluorinated alkyl substances (PFASs), 15 phthalates (PAEs), six phenols, and four parabens. Various statistical models for robustness tests and mediation analysis were used to explore associations with long-term amenorrhea and the role of serum globulin. Biological mechanisms were identified through an integrated strategy involving target analysis of key chemicals and long-term amenorrhea intersections, pathway analysis, and target validation. Results showed that women with long-term amenorrhea had higher exposure levels of Perfluorodecanoic acid, Perfluorohexane sulfonic acid (PFHxS), Perfluorononanoic acid, n-perfluorooctanoic acid (n_PFOA), n-perfluorooctane sulfonic acid, and Perfluoromethylheptane sulfonic acid isomers. Logistic regression with different adjustments consistently found significant associations between elevated PFAS concentrations and increased long-term amenorrhea risk, confirmed by Partial Least Squares Discriminant Analysis. Mediation analysis revealed that serum globulin partially mediated the relationship between PFAS exposure and long-term amenorrhea. Network and target analysis suggested that PFHxS and n_PFOA may interact with Signal Transducer and Activator of Transcription 3 (STAT3). This study highlights significant associations between PFAS exposure, particularly PFHxS and n_PFOA, and long-term amenorrhea, with serum globulin and STAT3 serving as mediators in the underlying mechanisms.

## 1. Introduction

Personal Care and Consumer Product Chemicals (PCCPs) encompass a wide array of substances commonly found in everyday items, including personal care products, household goods, and food packaging materials [1]. Notable examples include polyfluorinated alkyl substances (PFASs), phthalates (PAEs), phenols, and parabens. These chemicals are extensively used for their desirable industrial and commercial properties [2], such as flexibility enhancement, stability, and antimicrobial effects. However, many PCCPs are classified as endocrine-disrupting chemicals (EDCs), which interfere with hormonal signaling pathways. EDCs can disrupt hormone synthesis, secretion, metabolism, and receptor activity, thereby influencing essential physiological processes [3]. This interference is linked to a wide range of health issues, including reproductive health disorders, developmental abnormalities, immune dysfunction, and metabolic disturbances [4]. The ubiquity of PCCPs in daily life and their persistence in the environment have raised significant concerns about their potential health impacts, especially regarding their cumulative effects under mixed exposures [5]. Despite growing awareness, the full extent of their biological and health effects remains insufficiently explored.

Long-term amenorrhea, the absence of menstruation, is a common gynecological condition affecting approximately 3–5% of women annually [6]. Long-term amenorrhea, particularly when it occurs without clear underlying causes such as pregnancy or menopause, is often indicative of underlying endocrine or metabolic imbalances [7]. Symptoms associated with long-term amenorrhea range from irregular menstrual cycles and infertility to more severe complications such as osteoporosis and cardiovascular diseases [8]. The condition may also indicate systemic disruptions, including inflammation and altered lipid metabolism [9]. While long-term amenorrhea is widely recognized as a global health issue, its exact etiology remains poorly understood. Currently, no effective preventive strategies or standardized treatments have been established, highlighting the need for further research into its causes and contributing factors.

Emerging evidence suggests a potential link between PCCPs and the risk of long-term amenorrhea. Substances such as PFASs, PAEs, phenols, and parabens have been implicated in disrupting hormonal homeostasis, triggering inflammatory responses, and altering metabolic functions [4], all of which are mechanisms associated with long-term amenorrhea [10]. Studies have shown that elevated exposure to these chemicals may increase the risk of menstrual irregularities and reproductive health problems [11]. However, most existing research focuses on the effects of individual chemicals, leaving a critical knowledge gap regarding how mixed exposures to multiple PCCPs might collectively influence the risk of long-term amenorrhea. Understanding these interactions is essential for clarifying the potential health risks associated with widespread PCCP exposure.

The study of mixed chemical exposures and their impact on long-term amenorrhea is particularly important, given the multifactorial nature of the condition [4]. Key features of long-term amenorrhea include chronic inflammation, oxidative stress, and disruptions in lipid metabolism [12], all of which can contribute to its onset and progression. Mixed chemical exposures may exacerbate these pathological processes through complex and synergistic mechanisms, amplifying their impact on endocrine and metabolic pathways [13]. Importantly, serum globulin plays key roles in immunity, inflammation [14], and lipid metabolism [15]. Investigating the mediation role of serum globulin is essential for advancing our understanding of how PCCPs contribute to long-term amenorrhea and identifying potential targets for intervention.

Long-term amenorrhea can be associated with altered globulin levels. Studies have shown that disturbed globulin levels may contribute to the development of long-term amenorrhea by affecting hormonal balance [16]. Abnormal levels of serum globulin, which is involved in immune response and lipid metabolism, can indicate immune dysfunction or metabolic disorders [17,18]. Disrupted globulin levels are often associated with systemic inflammation, which can impair ovarian function, disrupt ovulation, and potentially lead to menopause [19,20].

Signal Transducer and Activator of Transcription 3 (STAT3) is a key transcription factor gene that belongs to the STAT family. STAT3 plays an important role in cellular signal transduction and transcriptional regulation, and is widely involved in biological processes such as immune responses, cell growth, differentiation, and inflammation [21,22]. Dysregulation of the STAT3 pathway alters hormonal balance, contributing to the development of amenorrhea [23].

This cross-sectional study aims to address these knowledge gaps by analyzing data from the National Health and Nutrition Examination Survey (NHANES) to explore the associations between individual and combined exposures to PFASs, PAEs, phenols, and parabens and the risk of long-term amenorrhea in a representative U.S. adult population. Furthermore, mediation analysis on serum globulin, along with the construction of target networks and molecular docking, was performed to provide deeper insights into the impact of key PCCPs on long-term amenorrhea.

## 2. Materials and Methods

### 2.1. Study Design and Population

Conducted annually by the National Center for Health Statistics (NCHS), part of the U.S. Centers for Disease Control and Prevention, NHANES combines detailed interviews with comprehensive physical exams to gather a wide range of health and nutrition information. This information is critical for assessing the health and nutrition status of the U.S. population [24]. The study was approved by the Research Ethics Review Board of the National Center for Health Statistics, and all participants provided written informed consent.

**Figure 1:**
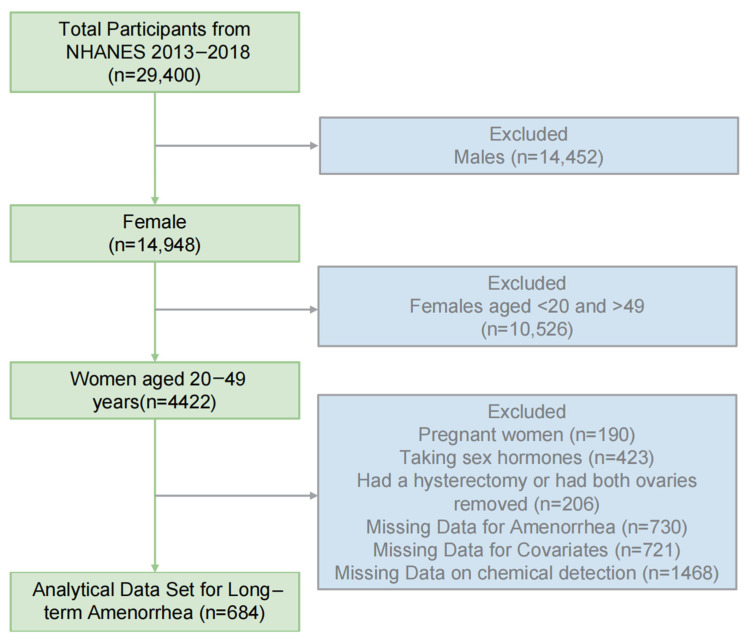
Flow chart of the screening process from NHANES (2013–2018).

We used data from NHANES, encompassing three survey cycles (2013–2014, 2015–2016, 2017–2018). Women aged 20–49 with complete data were included in the analysis, resulting in a final sample of 684 participants after applying the exclusion criteria. Participants with the following conditions were excluded [25], including (a) participants under 20 years of age or over 49 years of age, totaling 10,526; (b) 190 pregnant women; (c) 423 individuals taking sex hormones; (d) 206 individuals who underwent hysterectomy or bilateral oophorectomy; (e) 1451 with missing covariate or outcome data; and (f) 1468 individuals with missing chemical substance data. The exclusion process is shown in Figure 1.

### 2.2. Measurements of Chemical Exposure

Serum and urine samples were collected at mobile examination centers, stored at ≤−20 °C, and subjected to pretreatment. Specimens were processed, stored, and shipped to the Division of Laboratory Sciences, National Center for Environmental Health, National Centers for Disease Control and Prevention, Atlanta, Georgia [26]. Serum PFAS concentrations were measured by using online solid-phase extraction coupled to high-performance liquid chromatography–turbo ion spray ionization–tandem mass spectrometry. Urinary environmental chemical concentrations are determined by ICP-DRC-MS (inductively coupled plasma dynamic reaction cell mass spectroscopy) or detected using online solid-phase extraction, isotope dilution, and high-performance liquid chromatography separation, followed by electrospray ionization and tandem mass spectrometry. They were also tested in the same sample population between 2013 and 2018. Chemical concentrations in urine were adjusted for urinary creatinine to account for dilution. Abbreviations and detection details are provided in Text S1.

### 2.3. Measurement of Serum Globulin

Serum globulin (g/dL) was measured using the Beckman UniCel^®^ DxC800 Synchron instrument (Beckman Coulter Company, Shanghai, China) [27].

### 2.4. Assessment of Outcome Measures

Women were identified based on their responses to the question, “Have you had at least one period in the last 12 months?” Participants who answered “No” to this question and subsequently responded with “Other” or “Don’t know” to the follow-up question “What is the reason you have not had a period in the last 12 months?” were classified as having long-term amenorrhea [25]. Menstruating women were defined by women who answered “Yes” to the same question.

### 2.5. Covariates

Covariates included age, race, education, poverty income ratio (PIR), smoking status, alcohol consumption, and body mass index (BMI). These data were obtained from NHANES demographics, questionnaires, and physical examinations. Additional details are provided in Text S2.

### 2.6. Key Chemicals and Long-Term Amenorrhea Target Prediction

To obtain detailed information on the characterization and physicochemical properties of PFHxS and n_PFOA, the PubChem database (https://pubchem.ncbi.nlm.nih.gov/ (accessed on 1 November 2024)) was queried using the chemical identifiers CAS: 355-46-4 and 335-67-1 [28]. Target prediction was conducted using the SuperPred and SwissTarget platforms, utilizing Canonical SMILES notation for input. The predicted targets were subsequently mapped to corresponding gene names through bioDBnet (https://biodbnet-abcc.ncifcrf.gov/db/db2db.php (accessed on 1 November 2024)). These gene targets were then considered to be associated with the key chemicals. Additionally, the GeneCards database (https://www.genecards.org/ (accessed on 1 November 2024)) was consulted to identify relevant gene targets specifically linked to long-term amenorrhea.

### 2.7. Construction of Key Chemicals and Long-Term Amenorrhea Intersection Targets Network

Protein–protein interaction (PPI) networks for the identified intersection targets were constructed using the STRING database (https://cn.string-db.org/ (accessed on 8 November 2024)). The interaction data were downloaded in TSV format and imported into Cytoscape 3.10.2 for network visualization and further analysis.

### 2.8. GO and KEGG Enrichment Analyses

Gene Ontology (GO) enrichment and Kyoto Encyclopedia of Genes and Genomes (KEGG) pathway analyses were conducted for the target genes using R (v. 4.4.3). The selection of enriched terms was based on the clustering results provided by R.

### 2.9. Molecular Docking

Molecular docking simulations were performed using AutoDock Vina 1.1.2. The SDF files for the key chemicals were obtained from PubChem (https://pubchem.ncbi.nlm.nih.gov/ (accessed on 10 November 2024)), while the PDB files for human STAT3 (PDB codes: 1bg1) were retrieved from the Protein Data Bank (https://www.rcsb.org/ (accessed on 10 November 2024)). The binding affinities of chemicals to the target proteins were evaluated based on their binding energy values. The docking simulation results were visualized using PyMOL (version 1.2) and Discovery Studio (version 2.4).

### 2.10. Statistical Analysis

Sample weights were applied to account for the complex, multistage sampling design of NHANES. Various statistical models were used for robustness tests. As the concentrations of chemicals in blood and urine exhibited a skewed distribution, we used log-transformed values. Statistical differences in the covariates between the outcome groups were evaluated using a chi-squared test for categorical variables and a t-test for continuous variables. Logistic regression models with different adjustments were used to examine the associations between individual EDCs and the risk of long-term amenorrhea. Odds ratios (ORs) with corresponding 95% confidence intervals (CIs) were reported. We applied the Benjamini–Hochberg (BH) procedure for *p*-value correction. Partial Least Squares Discriminant Analysis (PLS-DA) was conducted to assess the effects of mixed exposures while controlling for confounding variables. Significant associations were identified by integrating the results from PLS-DA (Variable Importance in Projection (VIP) > 1) and logistic regression (*p* < 0.05) [29]. Mediation analyses were conducted to investigate whether the association between EDCs and long-term amenorrhea could be mediated by serum globulin.

## 3. Results

### 3.1. Study Population Baseline Characteristics

Table 1 lists the characteristics of the 684 women included in the study. No significant differences were observed between the two groups in creatinine levels, age, ethnicity, poverty-to-income ratio (PIR), education level, smoking status, or drinking status. However, a significant difference was observed in BMI (*p* = 0.026).

### 3.2. Exposure Levels of PFASs, PAEs, Phenols, and Parabens

As shown in Table 2, compared to the normal female group, the levels of PFDA, PFHxS, PFNA, n_PFOA, n_PFOS, and Sm_PFOS were significantly higher in the long-term amenorrhea group (all FC > 1 and *p* < 0.05). These results suggest that exposure to these six PFASs may increase the risk of long-term amenorrhea.

### 3.3. Logistic Regression and PLS-DA Analysis of the Association Between Selected Chemicals and Long-Term Amenorrhea Risk

Logistic regression analysis identified significant associations between selected chemicals and long-term amenorrhea. As detailed in Table 3, PFDA (OR: 1.70, 95% CI: 1.20–2.41), PFHxS (OR: 2.24, 95% CI: 1.54–3.26), PFNA (OR: 1.89, 95% CI: 1.17–3.05), n_PFOA (OR: 2.51, 95% CI: 1.71–3.70), n_PFOS (OR: 2.35, 95% CI: 1.43–3.86), and Sm_PFOS (OR: 3.24, 95% CI: 1.70–6.17) demonstrated significant positive correlations with the presence of long-term amenorrhea. To further validate these findings, PLS-DA was employed as a mixed exposure analysis method. This analysis confirmed the associations between PFDA, PFHxS, PFNA, n_PFOA, n_PFOS, and Sm_PFOS (all VIP > 1), demonstrating their significant contributions to the effects of mixed exposure on long-term amenorrhea. The FDR-corrected *p*_BH results remained largely unchanged, indicating the robustness of the findings.

To further explore the relationship between exposure to the six PFASs and long-term amenorrhea, we adjusted for covariates. The results of the three models were consistent, supporting the robustness of the findings. As presented in Table 4, even after fully adjusting for potential covariates in Model 3, significant associations with long-term amenorrhea were observed for PFDA (OR: 1.7, 95% CI: 1.17–2.48), PFHxS (OR: 2.35, 95% CI: 1.55–3.56), PFNA (OR: 1.78, 95% CI: 1.14–2.79), n_PFOA (OR: 2.30, 95% CI: 1.42–3.74), n_PFOS (OR: 2.46, 95% CI: 1.68–3.61), and Sm_PFOS (OR: 3.23, 95% CI: 1.82–5.73). It is worth mentioning that the *p*-values for PFHxS and n_PFOA, both before and after adjusting for covariates, were less than 0.001, indicating a strong positive correlation between both exposures and the risk of long-term amenorrhea.

### 3.4. Association Between Six PFASs’ Exposure, Serum Globulin as a Mediator, and the Outcome of Long-Term Amenorrhea

Given the importance of serum globulin and its diverse roles in chronic inflammation, oxidative stress, and lipid metabolism, for the six PFASs significantly associated with long-term amenorrhea, serum globulin was studied as a mediator. Interestingly, the direct and indirect effects of the PFASs’ exposure on long-term amenorrhea, with serum globulin as a mediator, were significant in PFHxS, PFNA, n_PFOA, and Sm_PFOS (all *p* < 0.05) (Table 5). Among the four PFAS exposures, serum globulin may play a partial role in mediating the risk of long-term amenorrhea in some cases, with an estimated mediation effect in approximately 10–20% of cases (PM: 10–22%) [30]. This suggests that serum globulin might be involved in the association between PFAS exposure and long-term amenorrhea risk.

### 3.5. Identification of Common Targets and PPI Network Construction

**Figure 2:**
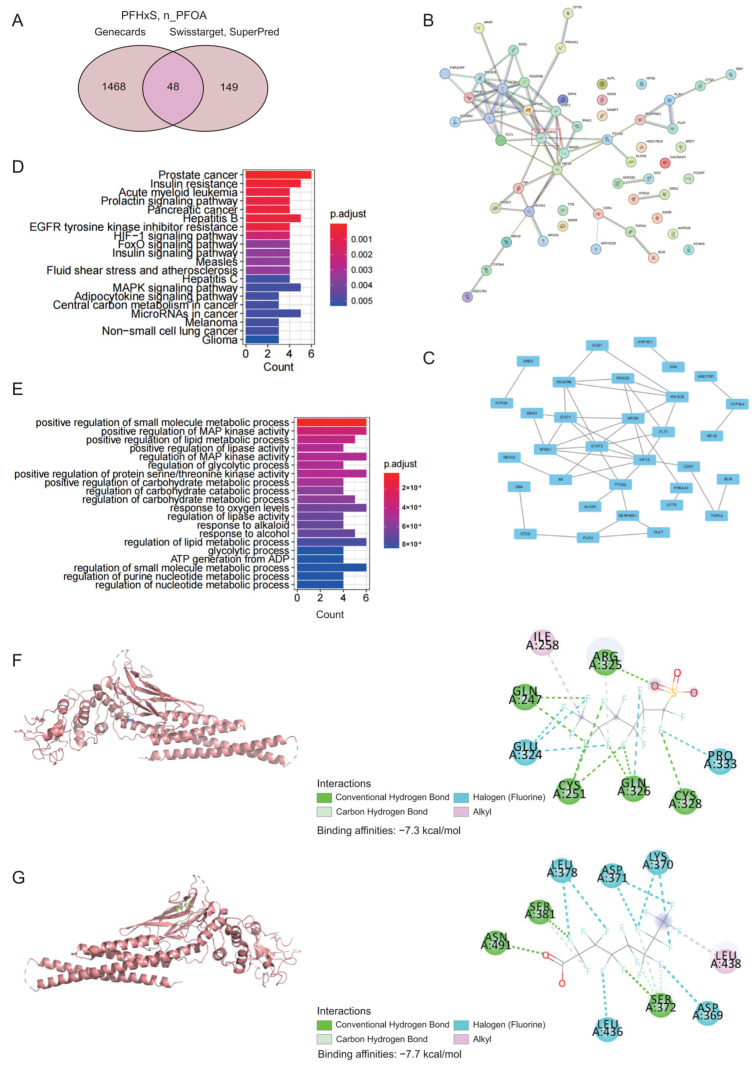
Identification of common targets, interaction networks, and target validation between PFHxS, n_PFOA, and long-term amenorrhea. (**A**) Venn diagram of common targets between PFHxS, n_PFOA, and long-term amenorrhea. (**B**,**C**) PPI network for PFHxS, n_PFOA, and long-term amenorrhea targets. (**D**,**E**) KEGG and GO pathway analyses of PFHxS and n_PFOA targets in long-term amenorrhea. (**F**,**G**) Molecular docking analysis of PFHxS and n_PFOA binding to STAT3 protein.

To explore the underlying mechanisms of PFASs in long-term amenorrhea, we selected the two most robust PFASs (PFHxS and n_PFOA) based on their highest VIP values (VIP > 2) [31], lowest *p*-values in all logistic models (*p* < 0.001), and significant mediation effects of serum globulin, utilizing SuperPred, SwissTargetPrediction, and GeneCards. Target prediction for PFHxS and n_PFOA targets was conducted using SuperPred and SwissTargetPrediction, identifying 197 targets. Using GeneCards, we predicted and identified 1516 targets related to long-term amenorrhea. A Venn diagram was generated by intersecting the PFHxS and n_PFOA targets with long-term amenorrhea-related targets, revealing 48 common targets (Figure 2A). Subsequently, a PPI network was constructed using STRING and visualized with Cytoscape 3.10.2. After excluding isolated targets, the final PPI network highlighted several critical interactions (Figure 2B,C), with STAT3 showing the highest degree of centrality.

### 3.6. GO and KEGG Enrichment Analyses

To explore the potential biological pathways and functions of PFHxS and n_PFOA in long-term amenorrhea, we performed GO and KEGG enrichment analyses on 48 common targets of PFHxS and n_PFOA using the R package clusterProfiler. KEGG pathway analysis and GO enrichment analysis (Figure 2D,E) for PFHxS and n_PFOA targets identified several key biological processes, including the regulation of small-molecule metabolic processes, positive regulation of lipid metabolic processes, and positive regulation of lipase activity. Interestingly, these processes are linked to serum globulin [15] and STAT3 [32].

### 3.7. Molecular Docking Analysis of PFHxS and n_PFOA Binding to Key Targets in Long-Term Amenorrhea

To validate the molecular interactions between PFHxS, n_PFOA, and the core target STAT3, we conducted molecular docking analysis using Autodock Vina 1.1.2. The threshold was set as −7 kcal/mol to consider the affinity of a chemical compound to its target protein as being strong [33]. The binding affinities for PFHxS-STAT3 and n_PFOA-STAT3 complexes were −7.3 kcal/mol and −7.7 kcal/mol, respectively, indicating strong binding interactions. Docking visualization revealed key hydrogen bond interactions in the PFHxS-STAT3 complex involving residues GLN247, CYS251, GLN326, CYS328, and ARG325. Halogen (fluorine) interactions were noted with GLU324 and PRO333, while alkyl interactions involved ILE258 (Figure 2F), suggesting a stable binding capacity for PFHxS with STAT3 [34]. For the n_PFOA-STAT3 complex, hydrogen bond interactions were identified with residues SER381, ASN391, and SER372, as well as halogen (fluorine) interactions with ASP371, LEU378, LYS370, LEU436, and ASP369. Additionally, alkyl interactions with LEU438 were observed, further supporting the binding stability of this complex [34] (Figure 2G).

## 4. Discussion

This study utilized population data from the NHANES database to compare normal reproductive-age women with those experiencing long-term amenorrhea. The results showed that, among PCCPs, the serum or urine levels of PFDA, PFHxS, PFNA, n_PFOA, n_PFOS, and Sm_PFOS were significantly higher in the disease group compared to the control group, suggesting that exposure to these six PFASs may be associated with the occurrence of long-term amenorrhea. Further analysis using logistic regression and PLS-DA revealed significant positive correlations between PFDA, PFHxS, PFNA, n_PFOA, n_PFOS, and Sm_PFOS and the presence of long-term amenorrhea. These PFASs also contributed significantly to the effects of mixed exposure on long-term amenorrhea. To validate the robustness of these associations, covariates were adjusted, and the results confirmed that the significant associations persisted, indicating the robustness of the findings. Notably, the *p*-values for PFHxS and n_PFOA consistently remained below 0.001 both before and after adjustment, indicating a strong positive correlation with the outcome variable. Additionally, PFHxS and n_PFOA exhibited the highest VIP values in the mixed exposure model, highlighting their primary roles in the risk of long-term amenorrhea. These findings align with existing evidence that PFHxS and n_PFOA disrupt endocrine signaling [35], which is crucial for maintaining regular menstrual cycles, and offer new insights into the effects of specific PCCPs on long-term amenorrhea under mixed exposure conditions.

Our study found that serum globulin partially mediates the relationship between exposure to PFASs (PFHxS, PFNA, n_PFOA, and Sm_PFOS) and long-term amenorrhea, highlighting a potential mechanistic link between protein-binding dynamics and reproductive health. Previous studies have shown that PFAS exposure is associated with changes in menstrual cycles and reduced fertility in women, likely due to its interference with hormonal balance [36,37,38]. Serum globulin may also be linked to long-term amenorrhea through mechanisms involving immune function, and lipid metabolism and its related endocrine disruption [39]. As an essential component of immunoglobulins, serum globulin is closely related to the body’s immune response [18]. Abnormal levels of serum globulin can indicate either excessive immune activation or suppression, which in turn can affect ovarian function [40]. High serum globulin levels are often associated with systemic inflammation [41], which, over time, may impair the ovarian reserve or disrupt ovulation [42]. Pro-inflammatory cytokines can alter the local ovarian microenvironment or inhibit hormone secretion, leading to menopause [43,44]. Additionally, oxidative stress related to inflammation can further damage ovarian cells [12]. Abnormalities in serum globulin levels can also reflect lipid metabolic disorders, which are a major risk factor for long-term amenorrhea [45]. Changes in serum globulin levels may indirectly indicate endocrine dysfunction, and are commonly associated with long-term amenorrhea [46]. Serum globulin also serves as a marker for the body’s nutritional status [26]. Low globulin levels may signal malnutrition and metabolic dysfunction, which can directly affect gonadal function and lead to long-term amenorrhea [47]. Additionally, GO and KEGG enrichment analyses indicate that the targets of PFHxS and n_PFOA significantly influence lipid metabolism and small-molecule metabolic processes [48]. PFASs disrupt lipid signaling and metabolic pathways, including fatty acid oxidation and lipoprotein metabolism, potentially resulting in energy imbalance and disorders in steroid hormone synthesis [49], ultimately affecting the menstrual cycle. Research suggests that PFAS exposure is associated with alterations in lipid and energy metabolism [50], further supporting the role of serum globulin and lipid metabolism as mediators.

Notably, the study identifies STAT3 as a critical molecular target linking PFHxS and n_PFOA exposure to reproductive dysfunction. STAT3 is a transcription factor activated by cytokines and growth factors that regulates processes such as cell proliferation, apoptosis, and inflammation [51]. The dysregulation of STAT3 is also associated with metabolic disorders, chronic inflammation, and hormonal imbalances [52], which may profoundly impact the mechanisms of long-term amenorrhea. Given the evidence that PFAS exposure may alter immune and endocrine functions [53], along with our findings regarding globulin as a mediator, this suggests a potential link between STAT3 and serum globulin as mediators in the occurrence of long-term amenorrhea [54]. Interestingly, through CTD analysis, we found that both STAT3 and its downstream target MTOR [55] were affected by n-PFOA and PFHxS. In addition, n-PFOA has been proven to cause dysregulation of the MTOR pathway [56,57]. The literature suggests that MTOR is related to metabolic disorders [58] and immune function [59], further suggesting that STAT3 may contribute to the occurrence of long-term amenorrhea through these mechanisms.

This study has several key strengths. It leveraged the NHANES database to uncover the association between mixed PCCP exposure and long-term amenorrhea, validating the findings through various statistical models. Additionally, through mediation analysis and a mechanistic exploration strategy based on toxicological networks and target validation, it provided valuable insights into the roles of serum globulin and STAT3 as mediators in the associations with key PFAS exposure.

However, the study has some limitations. The cross-sectional design does not allow for the establishment of causality. Furthermore, due to the limitations in data availability from NHANES, the effects of other PPCPs remain unexplored. The small number of amenorrhea cases is also a limitation. However, we employed various statistical methods, including logistic regression analysis, PLS-DA model, covariate adjustment, and weighted regression for robustness testing to improve the statistical robustness. Studies with a larger sample size of cases are still required in the future.

## 5. Conclusions

In conclusion, this study identified significant associations between exposure to PFDA, PFHxS, PFNA, n_PFOA, n_PFOS, and Sm_PFOS and long-term amenorrhea, with PFHxS and n_PFOA being the primary contributors to mixed exposure. Mediation analyses confirmed that serum globulin partially mediates the association between exposure to PFHxS, PFNA, Sm_PFOS, and n_PFOA and the risk of long-term amenorrhea. We found that the interactions between PFHxS and n_PFOA with STAT3 represent a potential underlying mechanism. These findings offer novel insights into the occurrence of long-term amenorrhea and support the potential of targeting PCCPs as preventive intervention measures. Future research should focus on validating these findings in diverse populations and deeply exploring the molecular mechanisms underlying the interactions between PFASs, serum globulin, and STAT3 signaling in the pathogenesis of long-term amenorrhea.

## Figures and Tables

**Table 1:** Population characteristics of individuals in NHANES 2013–2018 (n = 684).

Characteristic	Level/Classification	Normal Female	Long-Term Amenorrhea Female	*p*
Creatinine (Mean (SD))		134.82 (89.79)	112.91 (62.11)	0.161
Age (Mean (SD))		34.19 (8.14)	34.71 (9.99)	0.722
Race(%)				0.207
	Non-Hispanic White	75.85	85.29	
	Others	24.15	14.71	
BMI (%)				0.026 *
	Abnormal	66.31	64.71	
	Normal	33.69	35.29	
Smoking (%)				0.804
	Never smoked	66.77	64.71	
	Smoked	33.23	35.29	
Alcohol (%)				0.168
	No	10.15	2.94	
	Yes	89.85	97.06	
Education (%)				0.446
	High school and below	14.31	14.71	
	High school graduate/GED or equivalent	18.31	26.47	
	Some college or AA degree	39.38	35.29	
	College graduate or above	28.00	23.53	
PIR (Mean (SD))		1.10 (0.77)	1.24 (0.65)	0.323

Normal female (n = 650); long-term amenorrhea female (n = 34); *: *p* < 0.05.

**Table 2:** Distribution of PFAS, PAE, phenol, and paraben concentrations in the participants.

Classification	Chemical	Normal Female	Long-Term Amenorrhea	FC	*p*
		Median (Q1, Q3)	Female Median (Q1, Q3)		
PFASs	Perfluorodecanoic acid (PFDA)	0.10 (0.07, 14.80)	0.20 (0.07, 0.80)	2.000	0.002 *
	Perfluorohexane sulfonic acid (PFHxS)	0.60 (0.07, 10.80)	1.10 (0.20, 6.20)	1.833	<0.001 *
	2-(N-methylperfluoroctanesulfonamido)acetic acid (MPAH)	0.07 (0.07, 3.10)	0.07 (0.07, 0.80)	1.000	0.956
	Perfluorononanoic acid (PFNA)	0.40 (0.07, 6.00)	0.60 (0.20, 2.90)	1.500	0.023 *
	Perfluoroundecanoic acid (PFUA)	0.07 (0.07, 4.40)	0.10 (0.07, 0.50)	1.429	0.456
	n-perfluorooctanoic acid (n_PFOA)	1.00 (0.07, 9.10)	1.80 (0.30, 85.20)	1.800	<0.001 *
	n-perfluorooctane sulfonic acid (n_PFOS)	1.90 (0.07, 42.30)	3.20 (0.70, 12.70)	1.684	0.002 *
	Perfluoromethylheptane sulfonic acid isomers (Sm_PFOS)	0.70 (0.07, 9.80)	1.09 (0.20, 3.50)	1.557	<0.001 *
PAEs	Mono(carboxyisononyl) phthalate (MCNP)	1.90 (0.14, 876.40)	1.56 (0.20, 10.00)	0.821	0.937
	Mono(carboxyisoctyl) phthalate (MCOP)	9.00 (0.21, 691.40)	17.12 (0.60, 563.30)	1.902	0.65
	Mono(2-ethyl-5-carboxypentyl) phthalate (MECPP)	8.70 (0.28, 261.80)	6.88 (1.20, 31.90)	0.791	0.183
	Mono-n-butyl phthalate (MBP)	11.50 (0.28, 489.60)	8.40 (0.40, 38.90)	0.730	0.229
	Mono-2-hydroxy-n-butyl phthalate (MHBP)	0.90 (0.28, 94.30)	0.64 (0.28, 4.90)	0.711	0.734
	Mono(3-carboxypropyl) phthalate (MCPP)	1.30 (0.28, 2170.00)	1.62 (0.28, 33.10)	1.246	0.423
	Monoethyl phthalate (MEP)	34.35 (1.20, 102,452.00)	23.41 (2.90, 2614.40)	0.682	0.309
	Mono(2-ethyl-5-hydroxyhexyl) phthalate (MEHHP)	5.40 (0.28, 113.90)	4.51 (0.50, 26.10)	0.835	0.561
	Cyclohexane 1,2-dicarboxylic acid Monohydroxy isononyl ester (MHINCH)	0.400 (0.28, 79.70)	0.28 (0.28, 125.20)	0.700	0.721
	Mono(2-ethylhexyl) phthalate (MEHP)	1.100 (0.57, 50.50)	0.67 (0.57, 8.10)	0.609	0.688
	Mono-isobutyl phthalate (MiBP)	9.60 (0.57, 304.20)	6.53 (0.57, 31.20)	0.680	0.010 *
	Mono-2-hydroxy-isobutyl phthalate (MHiBP)	3.10 (0.28, 123.00)	2.36 (0.28, 17.20)	0.761	0.034 *
	Mono-isononyl phthalate (MiNP)	0.64 (0.64, 107.90)	0.64 (0.64, 64.90)	1.000	0.923
	Mono(2-ethyl-5-oxohexyl) phthalate (MEOHP)	3.50 (0.14, 64.00)	2.82 (0.30, 14.50)	0.806	0.453
	Mono-benzyl phthalate (MBzP)	4.80 (0.21, 340.10)	3.20 (0.30, 47.80)	0.667	0.977
Phenols	Benzophenone-3 (BP-3)	32.79 (0.28, 10,814.20)	20.88 (2.10, 2893.20)	0.637	0.754
	Bisphenol A (BPA)	1.20 (0.14, 792.00)	1.00 (0.14, 4.10)	0.833	0.013 *
	Bisphenol F (BPF)	0.23 (0.14, 115.20)	0.39 (0.14, 25.00)	1.696	0.819
	Bisphenol S (BPS)	0.50 (0.07, 67.50)	0.50 (0.07, 8.00)	1.000	0.294
	Triclocarban (TCC)	0.07 (0.07, 338.30)	0.07 (0.07, 0.50)	1.000	0.012 *
	Triclosan (TCS)	5.63 (1.20, 1610.00)	3.59 (1.20, 179.90)	0.638	0.625
Parabens	Butyl paraben (BP)	0.07 (0.07, 114.10)	0.07 (0.07, 32.70)	1.000	0.959
	Ethyl paraben (EP)	2.70 (0.71, 1893.80)	0.710 (0.710, 331.50)	0.263	0.784
	Methyl paraben (MP)	66.32 (0.71, 15,964.10)	101.40 (2.70, 573.90)	1.529	0.966
	Propyl paraben (PP)	11.90 (0.07, 1441.80)	22.20 (0.20, 227.60)	1.866	0.711

FC: Fold Change; *: *p* < 0.05.

**Table 3:** Associations between EDCs for personal care and long-term amenorrhea in NHANES 2013–2018.

Classification	Chemical	Number	OR (95% CI)	*p*	*p*_BH	VIP
PFASs	PFDA	661	1.70 (1.20, 2.41)	<0.01 *	0.03 *	1.29
	PFHxS	661	2.24 (1.54, 3.26)	<0.01 *	<0.01 *	2.01
	MPAH	661	0.87 (0.57, 1.33)	0.53	0.86	0.62
	PFNA	661	1.89 (1.17, 3.05)	0.01 *	0.07	1.37
	PFUA	661	1.17 (0.66, 2.08)	0.59	0.86	1.27
	n_PFOA	639	2.51 (1.71, 3.70)	<0.01 *	<0.01 *	2.06
	n_PFOS	639	2.35 (1.43, 3.86)	<0.01 *	0.01 *	1.62
	Sm_PFOS	639	3.24 (1.70, 6.17)	<0.01 *	0.01 *	1.89
PAEs	MCNP	677	0.99 (0.73, 1.35)	0.97	0.97	0.57
	MCOP	677	1.1 (0.80, 1.50)	0.56	0.86	0.77
	MECPP	677	0.79 (0.57, 1.10)	0.17	0.51	0.69
	MBP	677	0.86 (0.66, 1.13)	0.29	0.79	0.84
	MHBP	653	0.92 (0.66, 1.27)	0.6	0.86	0.73
	MCPP	677	1.15 (0.85, 1.54)	0.37	0.86	1.25
	MEP	677	0.9 (0.70, 1.16)	0.44	0.86	0.69
	MEHHP	677	0.91 (0.65, 1.28)	0.6	0.86	0.8
	MHINCH	677	1.14 (0.65, 2.00)	0.66	0.88	0.47
	MEHP	677	0.91 (0.52, 1.60)	0.75	0.92	0.53
	MiBP	677	0.69 (0.50, 0.95)	0.03 *	0.12	1.51
	MHiBP	653	0.79 (0.61, 1.03)	0.09	0.29	0.89
	MiNP	677	1.16 (0.72, 1.87)	0.54	0.86	0.79
	MEOHP	677	0.89 (0.65, 1.21)	0.46	0.86	0.79
	MBzP	677	1.03 (0.79, 1.33)	0.83	0.94	1
Phenols	BP-3	458	0.98 (0.72, 1.34)	0.9	0.97	0.05
	BPA	458	0.71 (0.54, 0.94)	0.02 *	0.11	0.65
	BPF	457	1.05 (0.70, 1.57)	0.83	0.94	0.32
	BPS	457	0.84 (0.59, 1.21)	0.36	0.86	0.49
	TCC	458	0.46 (0.23, 0.91)	0.03 *	0.13	0.76
	TCS	458	0.91 (0.64, 1.28)	0.58	0.86	0.44
Parabens	BP	458	0.94 (0.68, 1.31)	0.74	0.92	0.3
	EP	458	1.02 (0.73, 1.41)	0.92	0.97	0.13
	MP	458	0.99 (0.79, 1.25)	0.94	0.97	0.36
	PP	458	0.95 (0.76, 1.19)	0.67	0.88	0.31

CI: confidence interval; *: *p* < 0.05; *p*_BH represents the Benjamini–Hochberg-corrected *p* to control the false discovery rate; VIP: Variable Importance in Projection.

**Table 4:** Logistic regression analysis of the association between the 6 blood serum PFAS levels and long-term amenorrhea in the participants.

Chemical	Number	Model 1		Model 2		Model 3	
		OR (95% CI)	*p*	OR (95% CI)	*p*	OR (95% CI)	*p*
PFDA	661	1.70(1.20, 2.41)	0.005 *	1.67 (1.15, 2.43)	0.01 *	1.70 (1.17, 2.48)	0.008 *
PFHxS	661	2.24 (1.54, 3.26)	<0.001 *	2.27 (1.50, 2.45)	<0.001 *	2.35 (1.55, 3.56)	<0.001 *
PFNA	661	1.89 (1.17, 3.05)	0.013 *	1.79 (1.12, 2.86)	0.02 *	1.78 (1.14, 2.79)	0.016 *
n_PFOS	639	2.35 (1.43, 3.86)	0.002 *	2.22 (1.34, 3.68)	0.003 *	2.30 (1.42, 3.74)	0.002 *
n_PFOA	639	2.51 (1.71, 3.70)	<0.001 *	2.41 (1.68, 3.44)	<0.001 *	2.46 (1.68, 3.61)	<0.001 *
Sm_PFOS	639	3.24 (1.70, 6.17)	0.001 *	3.03 (1.65, 5.55)	0.001 *	3.23 (1.82, 5.73)	<0.001 *

CI: confidence interval; * *p* < 0.05; Model 1: crude (unadjusted); Model 2: adjusted race and age; Model 3: adjusted age, race, BMI, smoking, alcohol, education, and PIR.

**Table 5:** Mediation effects of PFAS exposures on long-term amenorrhea with serum globulin as the mediator.

Chemical	Estimated Indirect and Direct Effects				Estimated Proportion Effects			
	IE (95% CI)	*p*	DE (95% CI)	*p*	PM (95% Cl)	*p*	TE (95% Cl)	*p*
PFDA	0.005 (−0.008, 0.022)	0.418	0.083 (0.014, 0.181)	0.010 *	5.6% (1.4, 31.3)	0.414	0.089 (0.014, 0.190)	0.012 *
PFHxS	0.009 (0.002, 0.02)	0.008 *	0.085 (0.029, 0.160)	<0.001 *	10% (2.1, 31.3)	0.008 *	0.094 (0.037, 0.174)	<0.001 *
PFNA	0.017 (0.004, 0.038)	0.004 *	0.063 (0.001, 0.152)	0.048 *	22% (4.1, 90.3)	0.008 *	0.08 (0.015, 0.169)	0.004 *
n_PFOS	0.006 (−0.001, 0.017)	0.106	0.044 (0.021, 0.069)	<0.001 *	11% (−3.2, 31.9)	0.106	0.05 (0.024, 0.076)	<0.001 *
n_PFOA	0.014 (0.003, 0.029)	0.006 *	0.067 (0.031, 0.116)	<0.001 *	17% (3.5, 37.2)	0.006 *	0.081 (0.043, 0.134)	<0.001 *
Sm_PFOS	0.017 (0.003, 0.037)	0.014 *	0.142 (0.051, 0.265)	<0.001 *	10% (1.8, 26.4)	0.014 *	0.159 (0.063, 0.288)	<0.001 *

CI: confidence interval; * *p* < 0.05; DE: direct effect; IE: indirect effect; PM: estimated proportion mediated; TE: total effect. Models were adjusted for age, race, BMI, smoking, alcohol, education, and PIR.

## Data Availability

The original datasets supporting the analysis are publicly available on the NHANES website (https://www.cdc.gov/nchs/nhanes (accessed on 1 June 2024)). The analysis datasets and programming code are accessible from the corresponding author and will be provided if reasonably requested.

## Supplementary Materials

The following supporting information can be downloaded at: https://www.mdpi.com/article/10.3390/toxics13030187/s1, Text S1: Measurements of chemicals’ exposure; Text S2: Covariates.
