# Supplementary material for: Association of Personal Care and Consumer Product Chemicals with Long-Term Amenorrhea: Insights into Serum Globulin and STAT3
Source: Toxics. 2025 Mar 5;13(3):187. doi: 10.3390/toxics13030187 (PMC11945380; doi:10.3390/toxics13030187)
Supplement: Supplementary file 1 [file toxics-13-00187-s001.zip › toxics-3475291-supplementary.pdf]

Text S1. Measurements of chemicals exposure

S1.1. PFASs

Serum specimens are processed, stored, and shipped to the Division of Laboratory Sciences, National Center for Environmental Health, Centers for Disease Control and Prevention, Atlanta, GA for analysis. Detailed instructions on specimen collection and processing are discussed in the NHANES Laboratory Procedures Manual (LPM). Vials are stored under appropriate frozen (–20°C) conditions until they are shipped to National Center for Environmental Health for testing. The LLODs were 0.1 ng/mL for Perfluorodecanoic acid (PFDA), Perfluorohexane sulfonic acid (PFHxS), 2-(N-methylperfluoroctanesulfonamido)acetic acid (MPAH), Perfluorononanoic acid (PFNA), Perfluoroundecanoic acid (PFUA), n-perfluorooctanoic acid (n-PFOA), n-perfluorooctane sulfonic acid (n-PFOS) and Perfluoromethylheptane sulfonic acid isomers (Sm-PFOS).

S1.2. PAEs

Urine specimens are processed, stored, and shipped to the Division of Laboratory Sciences, National Center for Environmental Health, Centers for Disease Control and Prevention for analysis. Detailed specimen collection and processing instructions are discussed in the NHANES Laboratory Procedures Manual (LPM). Specimen vials are stored under appropriate frozen (–20°C) conditions until they are shipped to the National Center for Environmental Health for testing. The LLODs were 0.2 ng/mL for Mono(carboxyisononyl) phthalate (MCNP) and Mono(2-ethyl-5-oxohexyl) phthalate (MEOHP), 0.3 ng/mL for Mono(carboxyisoctyl) phthalate (MCOP) and Mono-benzyl

phthalate (MBzP), 0.4 ng/mL for Mono(2-ethyl-5-carboxypentyl) phthalate (MECPP), Mono(2-ethyl-5-hydroxyhexyl) phthalate (MEHHP), Mono-2-hydroxy-isobutyl phthalate (MHiBP), Mono-n-butyl phthalate (MBP), Mono(3-carboxypropyl) phthalate (MCPP), Mono(2-ethylhexyl) phthalate (MEHP), and Cyclohexane 1,2-dicarboxylic acid Monohydroxy isononyl ester (MHINCH), 0.8 ng/mL for Mono-2-hydroxy-n-butyl phthalate (MHBP), Monoethyl phthalate (MEP) and Mono-isobutyl phthalate (MiBP), 0.9 ng/mL for Mono-isononyl phthalate (MiNP).

S1.3. Phenols

Urine specimens are processed, stored, and shipped to the Division of Laboratory Sciences, National Center for Environmental Health, Centers for Disease Control and Prevention for analysis. Detailed specimen collection and processing instructions are discussed in the NHANES Laboratory Procedures Manual (LPM). Vials are stored under appropriate frozen (–20°C) conditions until they are shipped to National Center for Environmental Health for testing. The LLODs were 0.1 ug/L for Bisphenol S (BPS) and Triclocarban (TCC), 0.2 ug/L for Bisphenol A (BPA) and Bisphenol F (BPF), 0.4 ug/L for Benzophenone-3 (BP-3), 1.7 ug/L for Triclosan (TCS).

S1.4. Parabens

Urine specimens are processed, stored, and shipped to the Division of Laboratory Sciences, National Center for Environmental Health, Centers for Disease Control and Prevention for analysis. Detailed specimen collection and processing instructions are discussed in the NHANES Laboratory Procedures Manual (LPM). Vials are stored under

appropriate frozen (–20°C) conditions until they are shipped to National Center for Environmental Health for testing. The LLODs were 0.2 ug/L for Butyl paraben (BP) and Propyl paraben (PP), 1.0 ug/L for Ethyl paraben (EP) and Methyl paraben (MP).

Text S2. Covariates

Covariates in this study were grouped into three categories: Demographic, Lifestyle, and Reproductive Health-related.

Demographic covariates included age, race, education level, and the poverty income ratio (PIR), with data derived from NHANES demographic information. Race was classified as Hispanic or Non-Hispanic. Education was categorized into four levels: Less than High School, High School, Some College or Associate's Degree, and College Graduate or Above. PIR was categorized as low income (≤ 1), middle income (> 1 to ≤ 3), and high income (> 3).

Lifestyle covariates included smoking status, alcohol consumption, and body mass index (BMI). Smoking status was categorized into two groups: non-smokers and smokers, based on the response to the questions "Have you smoked at least 100 cigarettes in your lifetime?". Alcohol consumption was classified as either non-drinker or drinker. BMI was categorized into two groups: the normal group and the abnormal group. The normal group included individuals with a BMI between 18.5 kg/m^2 and 25 kg/m^2, while the abnormal group consisted of those who were underweight (BMI < 18.5 kg/m^2), overweight (BMI between 25 kg/m^2 and 30 kg/m^2), or obese (BMI ⩾ 30 kg/m^2). Data for these variables were obtained from body measures and questionnaire responses.

Reproductive health-related covariates included pregnancy status (determined by pregnancy test results), whether the individual takes sex hormones and whether they have had a hysterectomy or both ovaries removed. These data were collected through laboratory tests and the reproductive health questionnaire.
